# Nuclear Factor-Kappa B Inhibition Can Enhance Apoptosis of Differentiated Thyroid Cancer Cells Induced by ^131^I

**DOI:** 10.1371/journal.pone.0033597

**Published:** 2012-03-16

**Authors:** Zhaowei Meng, Shanshan Lou, Jian Tan, Ke Xu, Qiang Jia, Wei Zheng

**Affiliations:** 1 Department of Nuclear Medicine, Tianjin Medical University General Hospital, Tianjin, People's Republic of China; 2 Tianjin Normal University, Tianjin, People's Republic of China; 3 Tianjin Key Laboratory of Lung Cancer Metastasis and Tumor Microenviroment, Tianjin Lung Cancer Institute, Tianjin Medical University General Hospital, Tianjin, People's Republic of China; II Università di Napoli, Italy

## Abstract

**Objective:**

To evaluate changes of nuclear factor-kappa B (NF-κB) during radioiodine 131 (^131^I) therapy and whether NF-κB inhibition could enhance ^131^I-induced apoptosis in differentiated thyroid cancer (DTC) cells in a synergistic manner.

**Methods:**

Three human DTC cell lines were used. NF-κB inhibition was achieved by using a NF-κB inhibitor (Bay 11-7082) or by p65 siRNA transfection. Methyl-thiazolyl-tetrazolium assay was performed for cell viability assessment. DNA-binding assay, luciferase reporter assay, and Western blot were adopted to determine function and expression changes of NF-κB. Then NF-κB regulated anti-apoptotic factors XIAP, cIAP1, and Bcl-xL were measured. Apoptosis was analyzed by Western blot for caspase 3 and PARP, and by flow cytometry as well. An iodide uptake assay was performed to determine whether NF-κB inhibition could influence radioactive iodide uptake.

**Results:**

The methyl-thiazolyl-tetrazolium assay showed significant decrease of viable cells by combination therapy than by mono-therapies. The DNA-binding assay and luciferase reporter assay showed enhanced NF-κB function and reporter gene activities due to ^131^I, yet significant suppression was achieved by NF-κB inhibition. Western blot proved ^131^I could increase nuclear NF-κB concentration, while NF-κB inhibition reduced NF-κB concentration. Western blot also demonstrated significant up-regulation of XIAP, cIAP1, and Bcl-xL after ^131^I therapy. And inhibition of NF-κB could significantly down-regulate these factors. Finally, synergism induced by combined therapy was displayed by significant enhancements of cleaved caspase 3 and PARP from Western blot, and of Annexin V positively staining from flow cytometry. The iodine uptake assay did not show significant changes when NF-κB was inhibited.

**Conclusion:**

We demonstrated that ^131^I could induce NF-κB activation, which would attenuate ^131^I efficacy in DTC cells. NF-κB inhibition by Bay 11-7082 or by p65 siRNA transfection was effective in suppressing NF-κB regulated anti-apoptotic changes and in combined regimen apoptosis was achieved synergistically.

## Introduction

Thyroid nodule is a very common clinical problem and thyroid cancer is increasingly prevalent nowadays [1]. Differentiated thyroid cancer (DTC), including papillary and follicular thyroid cancer, comprises the majority of all thyroid cancers. Although the overall prognosis for DTC is good if total thyroidectomy and radioiodine 131 (^131^I) therapies are applied [2], patients with ^131^I-refractory metastases could only achieve a 10% 10-year survival rate [3], [4]. And for some cases, even ^131^I-avid lesions could not be successfully controlled by ^131^I therapy alone [5]. Therefore, development of novel anti-cancer methods is urgently needed for thyroid cancer.

In recent years, a number of culprit molecular targets have been identified in DTC carcinogenesis [6], [7], [8]. Among these, an emerging body of evidence shows that nuclear factor-kappa B (NF-κB) plays a crucial role in thyroid cancer, including cancer development and progression [6], [7], [9], [10], [11], [12], [13], [14]. It is also demonstrated that NF-κB induction by chemotherapy or radiotherapy could attenuate therapeutic efficacies. And NF-κB inhibition could promote thyroid cancer cell apoptosis, and to achieve synergistic effects [8], [9], [10], [11], [12]. However, to our knowledge, there has been no study investigating the relationship between NF-κB and ^131^I therapy in DTC, despite the importance of ^131^I treatment in DTC management.

Therefore, the purpose of the present research was to evaluate changes of NF-κB during ^131^I therapy. And we also aimed to determine whether combination with a NF-κB inhibitor or small interference RNA (siRNA) transfection could enhance ^131^I-induced apoptosis in DTC in a synergistic manner.

## Materials and Methods

### Cell culture

The human papillary thyroid carcinoma cell lines KTC-1, TPC-1 and follicular thyroid carcinoma cell line WRO were kindly provided by Dr. Shunichi Yamashita and Dr. Norisato Mitsutake (Department of Molecular Medicine, Atomic Bomb Disease Institute, Nagasaki University Graduate School of Biomedical Sciences, Nagasaki, Japan). DTC cells were grown in Dulbecco's minimum essential medium (GIBCO BRL, NY, USA) supplemented with 5% fetal bovine serum (GIBCO BRL, NY, USA), 1% (w/v) penicillin/streptomycin (Sigma-Aldrich, MO, USA) and 1 mU/mL thyrotropin (Sigma-Aldrich, MO, USA) in a 5% CO_2_ humidified atmosphere at 37°C.

### Transfection with siRNA

SMARTpool NF-κB p65 siRNAs was commercially designed by Dharmacon (Dallas, TX, USA). The pool of siRNAs contained the p65-specific sequences. Scrambled oligonucleotides (sequence AATTCTCCGAACGTGTCACGT) were chemically synthesized by SBS GeneTech (Beijing, China) and it was analyzed in a BLAST search to exclude homology to p65 or other genes [15]. When DTC cells were at 50%–60% confluence, transfection of p65 SMARTpool siRNAs (100 nmol/L), scrambled oligonucleotides (100 nmol/L) or no oligonucleotide (control) was performed using Lipofectamine 2000 (Invitrogen, Carlsbad, CA, USA) for 6 hours at 37°C in 6-well plates. Following transfection, cells were grown in new mediums and treated or collected as indicated.

### Methyl-thiazolyl-tetrazolium (MTT) assay

DTC cells (100 µl, 6000 cells per well) were seeded to 96-well plates and incubated for 48 hours before any treatment. Then new mediums were changed, and ^131^I (Beijing Atom HighTech, Beijing, China) or Bay 11-7082 (Sigma-Aldrich, MO, USA) were added in order to achieve final ^131^I activity concentrations of 5, 10, 20, 50, 100 and 200 MBq/ml or final Bay 11-7082 concentrations of 1, 2, 5, 10 and 20 µmol/L in each well. Cells were exposed to treatments for 48 hours, and 6 replicates were used for each concentration of either drug. In the control wells DMSO was added, and final concentration of DMSO did not exceed 0.2% in any well. After incubation, the cells were treated with MTT (50 µg/well, Sigma-Aldrich, MO, USA) for 1 hour at 37°C. The generated formazan was solubilized with 150 µl/well of DMSO, and the optical densities of the wells were measured at 450 nm with a Multiskan MS Plate Reader (Labsystems, Helsinki, Finland).

Proliferation of DTC cell lines after siRNA transfection was also measured by MTT assay. After transfection with p65 siRNA, scrambled oligonucleotides or no oligonucleotide control, cells were transferred as 6 replicates to 96-well plates at a concentration of 6000 cells per well. After 48 hours of incubation, cells were treated with or without 20 MBq/ml of ^131^I for 48 hours. Then MTT was performed as above.

### Preparation of cell extracts

After different treatments, cells were lysed in high salt buffer [9]. For total cell protein, harvested cells were suspended in 100 µl of lysis buffer (20 mM Tris-HCl pH 7.5, 1 mM EDTA, 150 mM NaCl, 0.5% Triton-X, 50 mM NaF, 10 mM sodium pyrophosphate, 1 mM sodium orthovanadate and 2 mM phenylmethyisulfonylfluoride) for 20 minutes on ice. Then lysate was centrifuged for 15 minutes at 14,000 rpm, and the supernatant was stored at −80°C until use. For nuclear protein, harvested cells were suspended in 400 µL of lysis buffer (10 mM HEPES pH 7.9, 10 mM KCl, 1.5 mM MgCl_2_, 0.5 mM dithiothreitol, 0.5 mM phenylmethylsulfonylfluoride and 0.1% Nonidet P-40) for 20 minutes on ice. Then lysate was centrifuged for 5 minutes at 14,000 rpm. The pellets were resuspended in 40 µL of nuclear extract buffer (20 mM HEPES pH 7.9, 420 mM NaCl, 1.5 mM MgCl_2_, 1 mM dithiothreitol, 0.2 mM EDTA, 0.5 mM phenylmethylsulfonylfluoride and 20% glycerol) for 20 minutes on ice. After centrifuge for 15 minutes at 14,000 rpm, the supernatant was collected and stored at −80°C until use. Protein concentrations were determined with a bicinchoninic acid assay reagent kit (Sigma-Aldrich, MO, USA).

### DNA-binding assay

The multi-well colorimetric assay for active NF-κB was performed [9]. Briefly, equal amount of nuclear extracts were incubated in 96-well plates coated with immobilized oligonucleotide containing a NF-κB consensus binding site. NF-κB binding to the target oligonucleotide was detected with primary antibody specific to p65 subunit and HRP-conjugated secondary antibody. For quantification of activity, optical densities were measured at 450 nm with a Multiskan MS Plate Reader.

### Luciferase assay

DTC cells were seeded in 24-well plates at 1×10^5^ cells per well. The cells were co-transfected with 400 ng of pNF-κB-luc (Clontech, Mountain View, CA, USA) and 4 ng of pRL-SV40 (Promega, Madison, WI, USA) using Lipofectamine 2000. The pRL-SV40 plasmid with a cDNA encoding *Renilla* luciferase was used as an internal control in each experiment [16]. Cells were rested for 12 hours after transfection, then incubated with or without ^131^I, or with combined therapy of ^131^I plus Bay 11-7082 for 6 hours. Activities of firefly and *Renilla* luciferases were determined sequentially from a single sample with the Dual-luciferase Reporter Assay system (Promega, Madison, WI, USA) using a Lumat LB 9507 luminometer (Bethold Technologies, Bad Wildbad, Germany).

DTC cells were co-transfected with pNF-κB-luc and pRL-SV40 at 24 hours after p65 siRNA or scramble transfection [15]. After 12 hours, the cells were treated with or without 20 MBq/ml of ^131^I for 6 hours. Then the reporter gene activities were assayed as described above.

### Western blot

Equal amounts of protein were electrophoresed by SDS-PAGE in 10% or 15% polyacrylamide gels. Proteins were transferred onto nitrocellulose membrane (Amersham Biosciences, NJ, USA) by semidry blotting. Membranes were blocked with Tris-buffered saline/0.1% Tween 20 (TBST) containing 5% milk for 60 minutes at room temperature, and then immune-blotted with appropriately diluted primary antibodies at 4°C overnight. After washing three times with TBST, the blots were incubated with HRP-conjugated secondary antibody for 60 minutes at room temperature. Then the complexes were visualized in iChemi XR imaging system (Syngene, MD, USA) by using chemiluminescence reagents (Millipore, MA, USA). Semi-quantification was performed by Quantity One Software Version 4.6.2 (Bio-Rad, CA, USA). Antibodies for NF-κB p65, X-linked inhibitor of apoptosis (XIAP), B-cell lymphoma extra large (Bcl-xL), cleaved caspase 3, poly-ADP-ribose polymerase (PARP) and β-actin were from Cell Signaling Technology (Beverly, MA, USA). Antibody for cellular inhibitor of apoptosis 1 (cIAP1) was from R&D Systems (Minneapolis, MN, USA). And antibody for proliferating cell nuclear antigen (PCNA) was from BD Transduction Laboratories (San Jose, CA, USA).

### Flow cytometry analysis of apoptotic cells

After 24 hours of different treatments, adherent cells were harvested by trypsinization, and 4×10^5^ cells were double stained with FITC-conjugated annexin V and propidium iodide for 15 minutes at room temperature in a Ca^2+^-enriched binding buffer (BD Pharmingen, CA, USA) and then subjected to FACSAria™ flow cytometer (BD Biosciences, CA, USA). FITC and propidium iodide emissions were detected in FL-1 and FL-3 channels, respectively. Analysis was done with Cell Quest software (BD Biosciences, CA, USA).

### Iodide uptake assay

To determine whether NF-κB inhibition can influence radioactive iodide uptake, iodide uptake assay was performed as described by Weiss et al. [17] with some modifications. KTC-1 cells were seeded in 6-well plates and treated with or without 5 µmol/L Bay 11-7082 for 24 hours. Then cells were cultured with 1 mL new medium per well, containing 37 KBq Na^125^I (Beijing Atom HighTech, Beijing, China), for 1 hour. Afterwards, the ^125^I-containing medium was decanted. Then cells were washed twice with PBS and trypsinized for total cell number counting. Finally radioactivity of the cells was measured with a γ counter (LKB 1261 Gamma Counter, Wallac, Turku, Finland).

To measure iodide uptake changes after siRNA transfection, KTC-1 cells transfected with p65 siRNA, scrambled oligonucleotides or no oligonucleotide control were transferred to 6-well plates. Then cells were incubated in the present of Na^125^I for 1 hour. Then cell number was counted, and radioactivity was measured.

### Statistical Analysis

All data were presented as mean ± SD. Statistics were performed with SPSS 15.0 (SPSS Incorporated, IL, USA). Differences between groups were analyzed by one-way analysis of variance (ANOVA) or independent samples *T*-Test. Least significant difference (LSD) test was used for multiple comparisons among groups. *P* value not exceeding 0.05 was considered statistically significant.

## Results

### Cytotoxic effect of ^131^I, Bay 11-7082 and siRNA therapies

**Figure 1:**
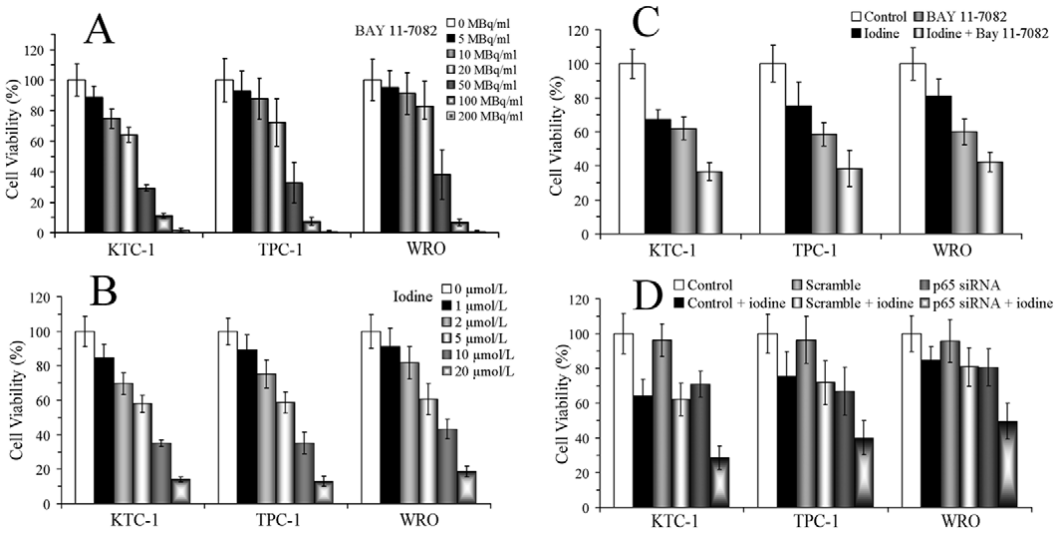
Cytotoxic effects of ^131^I, Bay 11-7082 and siRNA therapies on thyroid cancer cells. After exposure to indicated concentrations of Bay 11-7082 (A), activity concentrations of ^131^I (**B**) or combined therapies (**C**) for 48 hours, cell viabilities were determined by MTT assay. After DTC cells were treated with no oligonucleotide control, scrambled oligonucleotides or p65 siRNA transfection, cells were transferred to 96-well plates. Then cells were treated with or without ^131^I (20 MBq/ml) for 48 hours, and MTT was performed (**D**). Similar results were obtained in three independent experiments.

MTT assay was done after 48 hours of different treatments, and cell viabilities were calculated based on the total number of cells grown without any therapeutic intervention. As illustrated in Figure 1A and 1B, survival rates of all three cell lines showed inverse relationship to ^131^I activity concentrations and Bay 11-7082 dosage concentrations. For the following *in vitro* experiments, ^131^I activity concentration and Bay 11-7082 dosage concentration were determined as 20 MBq/ml and 5 µmol/L, respectively. Figure 1C showed that, by ANOVA, different treatment engendered different growth-inhibitory effects (*P*<0.01). And LSD revealed significant decrease of viable cells by combination therapy than by either mono-therapy (*P*<0.01).

Figure 1D showed that scrambled oligonucleotides transfection did not inhibit DTC cell proliferation, while siRNA transfection could achieve obvious inhibitory effects (*P*<0.05). Moreover, combination therapy could significantly induce stronger cell death than either ^131^I therapy or siRNA transfection (*P*<0.01).

### Effects of different therapies on NF-κB

**Figure 2:**
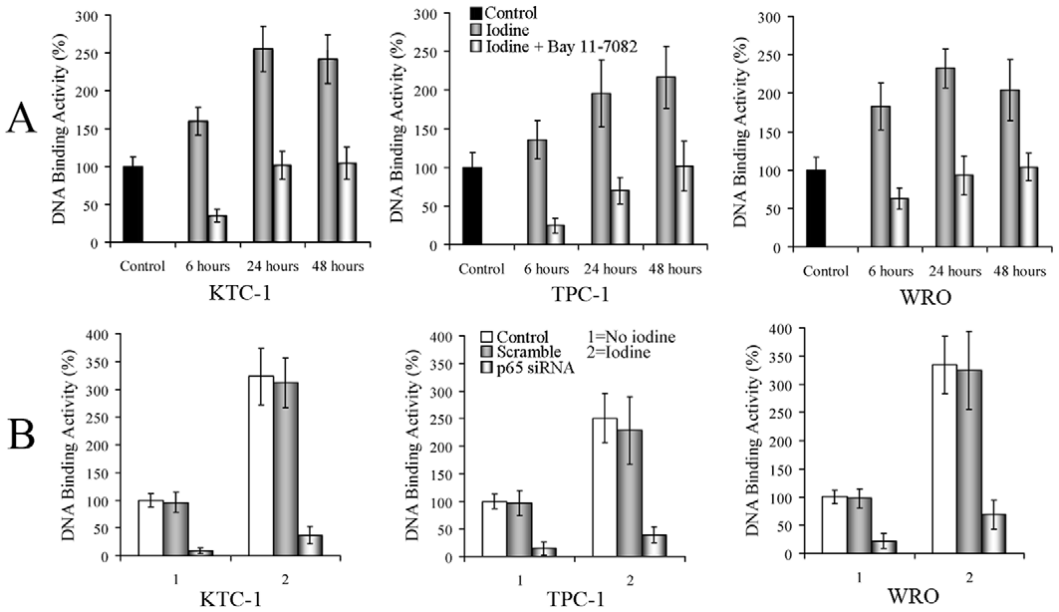
^131^I-induced NF-κB activation can be reduced by Bay 11-7082 or p65 siRNA treatments. (A) DTC cells were treated either with ^131^I (20 MBq/ml) or in combination with Bay 11-7082 (5 µmol/L) for 6, 24 and 48 hours. Nuclear extracts were prepared, and DNA-binding assay was done. (**B**) DTC cells were treated with no oligonucleotide control, scrambled oligonucleotides or p65 siRNA transfection. Then cells were exposed to ^131^I for 24 hours, and DNA-binding assay was performed. Similar results were obtained in three independent experiments.

To examine the effects of ^131^I on NF-κB, we performed DNA-binding assay using nuclear extracts from ^131^I-treated DTC cells for 6, 24 and 48 hours with untreated cells as control. NF-κB function increased during the time course of ^131^I mono-therapy, reaching peaks at 24-hour time point for KTC-1 and WRO cells. However, combination with Bay 11-7082 could significantly reduced NF-κB function (Figure 2A). In the above three time points, significant NF-κB inhibition was shown by *T*-Test in the combined therapies than ^131^I mono-therapies in all three cell lines (*P*<0.01). DTC cells were also transfected with p65 siRNA, scrambled oligonucleotides or no oligonucleotide control. Then three groups of all the cell lines were exposed to ^131^I for 24 hours. Although NF-κB function was strongly induced by ^131^I in both control and scramble groups, significant suppression could be obtained by p65 siRNA transfection (Figure 2B) in all three cell lines (*P*<0.01).

**Figure 3:**
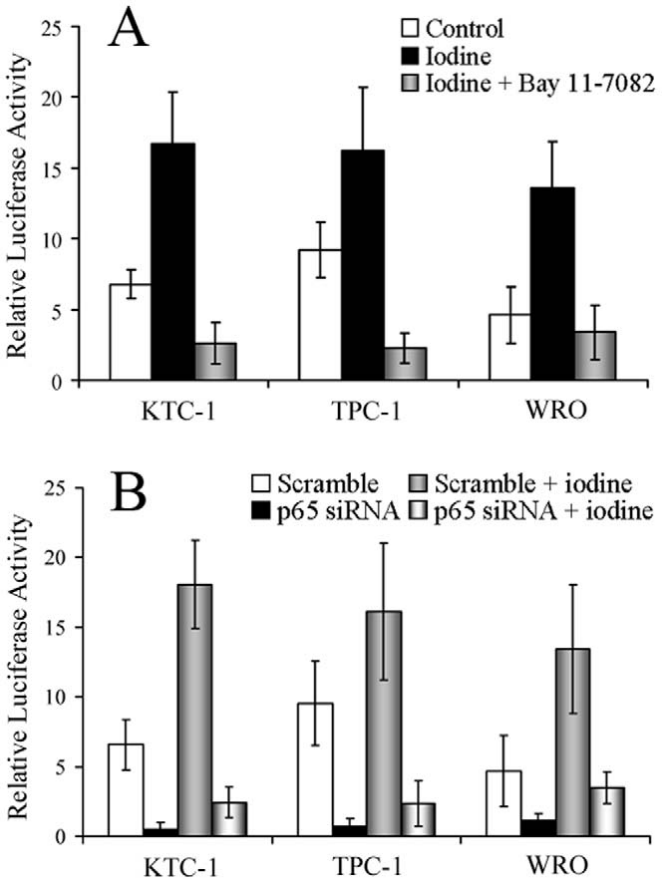
Repoter gene activity changes after different treatments. (**A**) DTC cells were transfected with pNF-κB-luc, and pRL-SV40 served as an internal control. After 12 hours, cells were left untreated or incubated with ^131^I (20 MBq/ml), or in combination with Bay 11-7082 (5 µmol/L) for 6 hours. NF-κB activation was detected by luciferase reporter assay. (**B**) At 24 hours after transfection with scrambled oligonucleotides or p65 siRNA, cells were co-transfected with pNF-κB-luc and pRL-SV40. After 12 hours, cells were left untreated or treated with ^131^I (20 MBq/ml) for 6 hours. Then luciferase reporter assay was performed. Similar results were obtained in three independent experiments.

NF-κB responsive promoter activity was performed as well (Figure 3). We found that ^131^I significantly enhanced the NF-κB reporter gene activities, to about 2.5, 1.7 and 3.0 folds in KTC-1, TPC-1 and WRO cell lines (*P*<0.05). Nevertheless, combination with Bay 11-7082 could inhibit NF-κB functions to about 0.16, 0.14 and 0.24 folds of the induced levels in KTC-1, TPC-1 and WRO cell lines (*P*<0.01). And p65 siRNA transfection could reduce NF-κB functions to nearly 0.14, 0.14 and 0.26 folds of the induced levels respectively (*P*<0.01).

**Figure 4:**
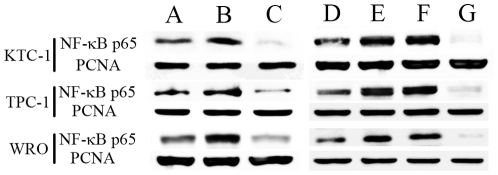
NF-κB protein level changes by different therapies. DTC cells were left untreated as controls (**A**), or treated with 20 MBq/ml ^131^I (**B**) or in combination with 5 µmol/L Bay 11-7082 (**C**) for 6 hours. Then nuclear protein extracts were examined for NF-κB p65 by Western blot. In the second set of experiments, control cells were left untreated (**D**). After no oligonucleotide (**E**), scrambled oligonucleotides (**F**) or p65 siRNA (**G**) transfection, DTC cells were treated with ^131^I (20 MBq/ml) for 6 hours. Then nuclear protein levels of NF-κB p65 were analyzed. PCNA was used as a loading control for nuclear proteins. Similar results were obtained in three independent experiments.

Next we examined nuclear NF-κB protein levels 6 hours after different treatments by Western blot (Figure 4). The amounts of p65 increased in ^131^I groups in all the cell lines, but suppressed obviously when Bay 11-7082 was added in the combination therapy. The similar results were obtained by p65 siRNA transfection, while scrambled oligonucleotides transfection could not achieve such effects.

### Effects of different therapies on NF-κB regulated anti-apoptotic factors

**Figure 5:**
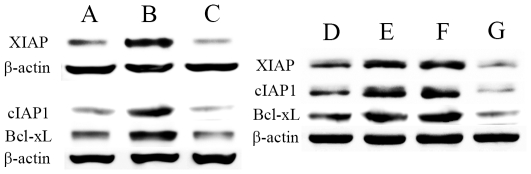
Effects of different treatments on NF-κB regulated anti-apoptotic factors. KTC-1 cells were left untreated as controls (**A**), or treated with 20 MBq/ml ^131^I (**B**) or in combination with 5 µmol/L Bay 11-7082 (**C**) for 24 hours. Then whole cell lysates were examined by Western blot for XIAP, cIAP1 and Bcl-xL. In the second set of experiments, control cells were left untreated (**D**). After no oligonucleotide (**E**), scrambled oligonucleotides (**F**) or p65 siRNA (**G**) transfection, KTC-1 cells were treated with ^131^I (20 MBq/ml) for 24 hours. Then protein levels of XIAP, cIAP1 and Bcl-xL were studied. β-actin was used as a loading control. Similar results were obtained in three independent experiments.

As being potent apoptosis inhibitors, XIAP, cIAP1 and Bcl-xL are target genes positively regulated by NF-κB [7], [18]. We tested what influences different treatments could inflict on them by Western blot, using KTC-1 as a representative cell line (Figure 5). Despite certain basal levels of XIAP, cIAP1 and Bcl-xL, ^131^I increased XIAP to 5.5–5.7 folds, cIAP1 to 4.8–6.7 folds and Bcl-xL to 3.6–5.1 folds, respectively. However, after combination with Bay 11-7082, XIAP, cIAP1 and Bcl-xL protein expressions were markedly reduced to 0.09, 0.10 and 0.17 folds of the enhanced levels respectively. After p65 siRNA transfection, these protein expressions were significantly inhibited to 0.07, 0.06 and 0.10 folds respectively. ANOVA and LSD revealed significant increases of these factors in ^131^I groups than in control groups (*P*<0.01), and significant decreases of them in combination groups than in ^131^I groups (*P*<0.01).

### Synergistic effects detected in combination therapies

**Figure 6:**
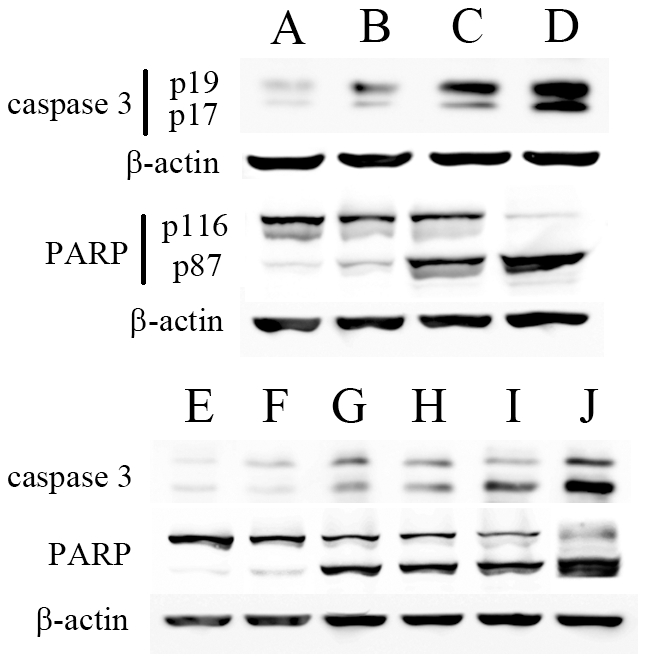
Effect of different treatments on apoptotic cascade. KTC-1 cells were left untreated as controls (**A**), or treated with 20 MBq/ml ^131^I (**B**), 5 µmol/L Bay 11-7082 (**C**) or combination (**D**) for 24 hours. Then whole cell lysates were examined by Western blot for caspase 3 and PARP. In the second set of experiments, KTC-1 cells were treated with no oligonucleotide control (**E**), scrambled oligonucleotides (**F**) or p65 siRNA transfection (**G**) first. Then these three groups of cells were exposed to ^131^I (20 MBq/ml) for 24 hours (**H–J**). Caspase 3 and PARP protein levels were examined by Western blot. β-actin was used as a loading control. Similar results were obtained in three independent experiments.

**Figure 7:**
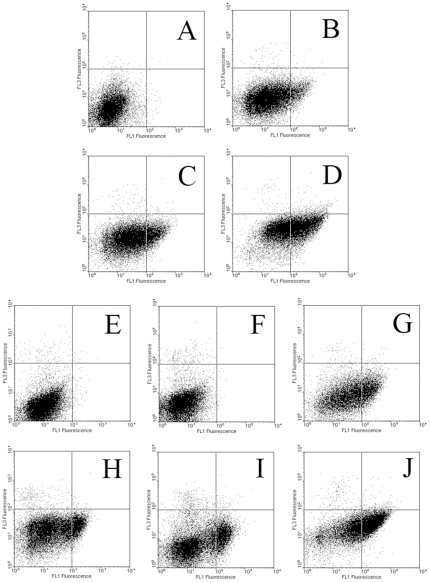
Apoptotic changes measured by flow cytometry. The first set of experiments included four groups: KTC-1 cells received no treatment (**A**), 20 MBq/ml ^131^I (**B**), 5 µmol/L Bay 11-7082 (**C**) or combination (**D**) for 24 hours. Then cells were harvested by trypsinization, double stained by FITC-conjugated annexin V and propidium iodide, and then subjected for flow cytometry. In the second set of experiments, KTC-1 cells were first treated with no oligonucleotide control (**E**), scrambled oligonucleotides (**F**) or p65 siRNA transfection (**G**). Then these three groups of cells were exposed to ^131^I (20 MBq/ml) for 24 hours (**H–J**). And flow cytometry was conducted. Similar results were obtained in three independent experiments.

First, Western blot was applied to detect changes of caspase 3 (a key apoptosis executioner) and PARP (a main target of caspase 3). We showed that although any intervention could induce cleavages of caspase 3 (subunits p19 and p17) and PARP (p89), their levels increased significantly further by combined treatments (Figure 6). In Table 1, ANOVA and LSD showed significant enhancements in combined therapies than in mono-therapies (*P*<0.05). Then, to further confirm the effects on apoptosis, double staining flow cytometry was performed. Results showed that while any therapy could induce apoptosis, combined treatments increased apoptosis synergistically due to significant enhancements of Annexin V positively stained cells (Figure 7 and Table 1).

**Table 1:** Synergy confirmed by Western blot and flow cytometry.

	Western blot with semi-quantification*				Flow cytometry
	Caspase 3 p19	Caspase 3 p17	PARP p89	PARP p116	Annexin V positive cells
Control	4.72±0.69	4.46±0.55	4.65±0.70	93.02±13.44	0.36±0.18
^131^I_(1)_	25.68±6.35	19.64±3.89	32.70±7.93	64.12±10.14	9.96±2.01
Bay 11-7082_(2)_	59.53±8.55	41.29±8.51	74.89±11.90	45.68±8.33	19.43±4.29
Combined therapy_(3)_	103.74±12.41	92.41±10.97	101.53±15.78	3.84±0.55	49.95±6.95
*F* (*P*)**	83.79 (<0.01)	85.06 (<0.01)	49.22 (<0.01)	47.43 (<0.01)	78.15 (<0.01)
*P*_(1) ∶ (3)_ ***	<0.01	<0.01	<0.01	<0.01	<0.01
*P*_(2) ∶ (3)_ ***	<0.01	<0.01	<0.05	<0.01	<0.01

* calculated by protein/β-actin with Quantity One Software;

** analyzed by one-way analysis of variance;

*** analyzed by least significant difference test.

### Iodide uptake in DTC cells under NF-κB blocking

**Figure 8:**
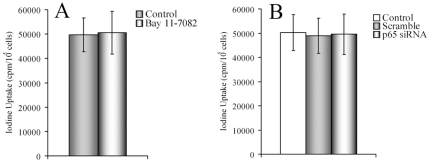
Iodide uptake assay. (A) KTC-1 cells were left untreated or treated with Bay 11-7082 (5 µmol/L) for 24 hours. Then cells were cultured in the present of Na^125^I (37 KBq/mL) for 1 hour. Afterwards, the cells were washed and cell number was counted. Then radioactivity was measured. (**B**) KTC-1 cells were treated with no oligonucleotide control, scrambled oligonucleotides or p65 siRNA transfection first. Then cells were exposed to Na^125^I, and radioactivity was measured. Similar results were obtained in three independent experiments.

To determine whether NF-κB pathway inhibition influences iodine uptake, we tested radioactive iodine uptake changes with or without NF-κB inhibition by Bay 11-7082 or p65 siRNA transfection. Figure 8 demonstrated that there were only slight changes after therapeutic interventions, no significant differences were observed (*P*>0.05), which indicated that NF-κB did not control iodine uptake in KTC-1 cells.

## Discussion

The most effective post-surgical treatment of DTC lesions is ^131^I therapy, due to the inherent ability of thyroid cancer cells to concentrate iodine. ^131^I emits short pathlength (0.5–2.2 mm) β-radiation that is cytotoxic to the cells, which is intrinsically very similar to external radiotherapy. However, this methodology is ineffective in patients whose tumors no longer concentrate ^131^I efficiently [4], [19], [20]. We tested our hypothesis in this study, that NF-κB could be an important factor regulating the anti-apoptotic process during ^131^I therapy. NF-κB has been shown to play an important role in many types of cancer [14] including thyroid cancer [7], [8], [13], [21], [22], which heralded searches for drugs capable of suppressing NF-κB activity [7], [18]. Till now, a number of NF-κB inhibitors have been demonstrated to induce thyroid cancer cell apoptosis, for instance, SN50, DHMEQ, bortezomib and Bay 11-7082. And when they were used in combination with chemotherapy or radiotherapy, synergism could be achieved [9], [10], [11], [12]. Another approach of p65 siRNA transfection has been tried in many types of cancers [15], [23], [24], [25], and enhanced chemosensitivity was demonstrated as well [15], [23]. Yet, combined therapy including p65 siRNA transfection has not been tested in thyroid cancer so far.

In the current study, the effects of ^131^I on NF-κB function and expression in DTC cells were analyzed first. DNA-binding assay showed that NF-κB activation was dramatically increased at 6-hour time point, reaching peaks at 24-hour time point for KTC-1 and WRO cells. Increased NF-κB reporter activation was also proved by luciferase assay. However, these activations could be significantly suppressed by a NF-κB inhibitor or by p65 siRNA transfection. We then used Western blot data to show that ^131^I could increase nuclear protein levels of NF-κB, while Bay 11-7082 or p65 siRNA transfection could dramatically inhibit NF-κB protein levels.

NF-κB mediated anti-apoptosis are essentially depended on its ability to enhance transcriptions of cell death suppressive genes [7], [18], [26]. The most representative NF-κB controlled anti-apoptotic factors are XIAP, cIAP1 and Bcl-xL, which can prevent tumor cell killing effects [7], [18]. XIAP can inhibit caspase 3 and 7, and it is also involved in the suppression of the pro-apoptotic JNK activity. cIAP1 is another potent inhibitor of apoptosis, which can bind and inhibit caspase 3 and 8. And Bcl-xL is known to inhibit cytochrome C release from mitochondria. In this investigation, we found that XIAP, cIAP1 and Bcl-xL were significantly up-regulated after exposure to ^131^I. Inhibition of NF-κB by Bay 11-7082 or by p65 siRNA transfection during ^131^I exposure could change the balance toward significant down-regulation of these factors.

Then, in order to confirm the possibility of synergistic effects induced by combined therapies, we performed Western blot on key apoptotic proteins and conducted flow cytometry to assess apoptosis. Our results demonstrated significant synergy if NF-κB was inhibited during ^131^I therapy. Our study also showed that NF-κB pathway inhibition did not control iodine uptake, which indicates its pro-apoptotic effects is the main mechanism for the synergistic results.

Apoptosis is an essential process of eliminating destined cells during development or after damage from chemotherapy or radiotherapy. It is known that ^131^I-induced cell death can be both apoptosis and necrosis, the nature of which is dose-dependent [27]. Marx et al. [27] demonstrated that, in B-CPAP cells (another DTC cell line), apoptosis was detectable after incubation with 1 MBq/ml of ^131^I for 2 days. At medium ^131^I activity concentration (1–10 MBq/ml) apoptosis was predominant, while at much higher ^131^I activity concentration (especially higher than 100 MBq/ml) necrosis became predominant. In our investigation, we used ^131^I activity concentration of 20 MBq/ml, which could produce enough apoptosis for different experiments. The slight dosage difference could be from different cell lines and different laboratory conditions.

Destruction of pro-apoptotic or anti-apoptotic balance is proved to cause carcinogenesis. In cancer treatment settings, aberrant apoptotic signaling could induce resistance to chemotherapy or radiotherapy. Therefore, restoration of functional apoptosis in cancer cells could be a useful approach to enhance therapeutic efficacies. NF-κB pathway hypothesis has been tested true in thyroid cancer already [9], [10]. Increased NF-κB activity reinforces the intrinsic therapy-resistance phenotype of thyroid cancer cells, and its inhibition has been showing promising results in combination with chemotherapy or radiotherapy [9], [10]. The present data adds to the hypothesis, that ^131^I could induce NF-κB activation which attenuates ^131^I efficacy in thyroid cancer cells. NF-κB inhibition is effective in suppressing ^131^I related NF-κB induction and anti-apoptotic changes. In combined regimen apoptosis can be achieved synergistically.
